# Redox unbalance in the hyperthyroid cat: a comparison with healthy and non-thyroidal diseased cats

**DOI:** 10.1186/s12917-019-1896-7

**Published:** 2019-05-08

**Authors:** Alessia Candellone, Paola Gianella, Lara Ceccarelli, Graziella Raviri, Paola Badino, Silvia Roncone, Hans S. Kooistra, Giorgia Meineri

**Affiliations:** 1 Department of Veterinary Science, University of Turin, L. go P. Braccini 2-5, 10095 Grugliasco, TO Italy; 2 Istituto Zooprofilattico Sperimentale of Piedmont, Liguria and Valle d’Aosta, V. Bologna 148, 10148 Turin, Italy; 3 Ambulatorio Veterinario “Antica Reggia” dott.ssa G. Raviri, P.zza V. Veneto 3, 10078 Venaria Reale, TO Italy; 4 Department of Clinical Sciences of Companion Animals, Faculty of Veterinary Medicine, Utrecht University, Yalelaan 108, 3584 CM Utrecht, The Netherlands

**Keywords:** Feline hyperthyroidism, Redox unbalance, Oxidative stress, Antioxidant status

## Abstract

**Background:**

Feline hyperthyroidism, the most common endocrinopathy in older cats, provides a spontaneous model for human thyrotoxicosis. Human thyrotoxicosis is associated with redox unbalance, which may result in organ damage. The redox status of hyperthyroid cats is largely unknown. The aims of the present study were to compare the redox status of cats with hyperthyroidism with that of healthy cats and cats with chronic non-thyroidal illness.

**Results:**

Forty cats with untreated hyperthyroidism (group H), 45 chronically ill cats with non-thyroidal illness (group I), and 39 healthy cats (group C) were recruited for this observational cross-sectional study. All cats were screened for redox status markers. Determinable reactive oxygen metabolites (d-ROMs) were used as oxidative stress markers. Antioxidant status was determined using the OXY-Adsorbent test to quantify the plasma barrier to oxidation. The Oxidative Stress index (OSi) was calculated as the ratio of d-ROMs and OXY-Adsorbent test values**.** Data were compared by ANOVA with Tukey’s multiple comparisons post-hoc test. The dROMs of group H (193 ± 47 CarrU) were significantly higher (*p* &lt; 0.001) than those of the healthy cats (103 ± 17 CarrU). The OXY-Adsorbent test results in group H (265 ± 68 μmol HClO/ml) were significantly lower than those in healthy cats (390 ± 83 μmol HClO/ml; *p* &lt; 0.01) and chronically ill cats (306 ± 45 μmol HClO/ml, *p* &lt; 0.05). Moreover, the Osi value in group H (0.8 ± 0.2 CarrU/μmol HClO/ml) was significantly higher (*p* &lt; 0.001) than that of the healthy cats (0.3 ± 0.1 CarrU/μmol HClO/ml).

**Conclusions:**

As described in humans with hyperthyroidism, feline hyperthyroidism is associated with redox unbalance. Free radical production is increased in hyperthyroid cats and their antioxidant depletion seems to be more severe than in cats with non-thyroidal illnesses. Our results support the rationale for a clinical trial investigating the potential positive effects of antioxidant supplementation to cats with hyperthyroidism.

**Electronic supplementary material:**

The online version of this article (10.1186/s12917-019-1896-7) contains supplementary material, which is available to authorized users.

## Background

Feline hyperthyroidism (FH) is the most common endocrinopathy in middle-aged and geriatric cats [1, 2]. Hyperthyroid cats and humans share clinical, pathological, and therapeutic features. Feline hyperthyroidism most often results from benign adenomatous nodules of the thyroid tissue, making it pathologically similar to Plummer’s disease (toxic nodular goitre) in humans. It also resembles Basedow-Graves’ disease in clinical appearance and therapy [3].

In hyperthyroid cats, pharmacotherapy with thyroid peroxidase inhibitors, so-called anti-thyroid drugs, is often the sole treatment option when radioiodine therapy is unavailable or when concurrent geriatric problems are likely to increase the risk of anesthesia-related complications of thyroidectomy [4]. Side-effects are a well-known complication in cats treated with anti-thyrotoxic agents [5, 6].

Redox unbalance, defined as a disturbance in the balance between the production of free radicals (oxidative stress) and antioxidant defences (antioxidant status), is well documented in human patients and experimental animals with hyperthyroidism [7–9]. Metabolic oxidation (associated with the hypermetabolic state) has been postulated as the origin of signs and symptoms of hyperthyroidism in humans [10–13]. Furthermore, redox unbalance is considered a risk factor for idiosyncratic drug toxicity syndromes in both humans and animal models [14–16]. Studies on hyperthyroid patients show that oxidative stress markers normalize after treatment and return to a euthyroid state [10, 16], while the occurrence of drug-related side-effects can be reduced with the concurrent administration of antioxidants [12, 13].

Redox unbalance has been detected in various illnesses in cats, including liver disease [17], FIV infection [18], chronic kidney disease [19–22], and cardiac disease [23]. Studies investigating oxidative stress and antioxidant status in hyperthyroid cats are scarce [24, 25].

The level of redox unbalance can be estimated by determining markers for oxidative stress and antioxidant status. Determinable reactive oxygen metabolites (d-ROMs) can be used as an indicator of free radical production and as a marker for oxidative stress, as well. Antioxidant status can be determined using the OXY-Adsorbent Test to quantify the plasma barrier to oxidation. The Oxidative Stress index (OSi), a measure that takes into account both oxidative stress and antioxidant status, is calculated by the ratio of the d-ROMs test result to the OXY-Adsorbent test result [26, 27].

The aims of the present study were to evaluate the redox status in FH and to compare the oxidative risk of the hyperthyroid population to that of healthy cats and cats with chronic non-thyroidal illness.

## Methods

### Cats

The study was approved by the local ethical committee and a written informed consent was obtained from all cat owners. For this observational cross-sectional study, cats presented to the Veterinary University Hospital of the Department of Veterinary Science (University of Turin, Italy) and other partner clinics and practices throughout northern Italy, from November 2016 to September 2017, were allocated in three groups. Group H comprised cats with spontaneous, untreated hyperthyroidism. Group I included cats with chronic non-thyroidal illness. Group C was composed of healthy cats. Only indoor cats older than 6 years of age (i.e. mature, senior and geriatric cats) were included in the study. The cats were categorized as hyperthyroid, chronically ill or healthy according to their history, physical examination, results of complete blood biochemical profile, urinalysis, serum total thyroxine (TT4) concentration, thoracic radiographic findings, and abdominal and/or cardiac ultrasound when deemed necessary for diagnosis. Body condition score (BCS) was measured according to the 1–9 WSAVA point-scale [28]. Chronically ill cats had to be newly-diagnosed with an infectious/inflammatory conditions, a metabolic diseases, a neoplasia or with chronic kidney disease (CKD, IRIS Stage ≥2) and had to show clinical signs for at least 3 weeks [29]. Hyperthyroid cats with a concurrent systemic disease such as congestive heart failure, symptomatic renal failure (IRIS Stage 3 or 4, creatinine &gt; 2.9 mg/dL; [29]), systemic neoplasia, chronic liver disease, immune-mediated disease, or systemic infection, any of which could influence antioxidant status independently of thyroid status, were excluded from this study; as were cats treated with antioxidants and/or methimazole within the last 3 months, fed with iodine-restricted food or a commercial diet enriched with patented antioxidant formula, or suspected of having nutritional deficiencies or excesses.

### Analytic procedures and redox balance assessment

All cats were fasted 12 h prior to blood sampling. Hematology tubes containing EDTA were stored at + 4 °C and analyzed the same day to obtain red blood cells (RBC), white blood cells (WBC), hematocrit (Hct), and hemoglobin (Hb) values. Tubes without anticlotting agents were immediately centrifuged at 1500 g for 10 min. Serum was divided into two aliquots of a minimum 0.5 ml each and stored in plastic flasks resistant to freezing at − 20 °C until processing. The first aliquot was used for biochemical analysis (albumin, ALB; blood urea nitrogen, BUN; creatinine, CREA; alanine-amino transferase, ALT; glucose) and TT4; the second aliquot used to assess the redox status, was processed within 3 months [27, 30]. TT4 concentrations were measured by chemiluminescence (Catalyst Total T4 assay run on the IDEXX Catalyst One analyse). TT4 &gt; 54 nmol/L or 4.3 μg/dL was considered consistent with hyperthyroidism. Determinable reactive oxygen metabolites (d-ROMs) were quantified using the d-ROMs Test (Diacron International Srl, Grosseto, Italy) as an indicator of oxidative stress due to free radicals. Antioxidant status was estimated using the OXY-Adsorbent Test (Diacron International) to quantify the plasma barrier to oxidation. The Oxidative Stress index (OSi) was calculated by the ratio of the d-ROMs test result to the OXY-Adsorbent test result [25–27, 30]. The assays used in this study had been previously used in cats by Castillo et al. [30], but data about their validation in the feline species were not available. A validation study for the use of d-ROMs test and OXY-Adsorbent test on cat sera was performed, according to a protocol modified from Pasquini et al., [31]. The within-run precision was estimated by calculating the intra-assay Coefficient of Variation (CV) on the basis of the results obtained after performing the tests in 12 samples repeated 3 times. Between-run precision was evaluated by assessing the inter-assay CV using the results obtained from 12 samples repeated 2 times. The linearity was assessed using two serum samples, the first one for d-ROMs test (264 Carr U) and the second one for OXY-Adsorbent test (473 μmol HClO/ml). Samples were diluted 1:1, 1:2, 1:4 and 1:8 in bi-distilled water and tested. A correlation analysis was used to examine the relationship results of diluted samples and expected results and the Coefficient of Regression (R^2^) was calculated.

Moreover, in order to confirm stability of dROMs and OXY-Adsorbent values during long-term storage at − 20 °C [27, 30] a validation study was performed. Briefly, 7 cat samples were utilized to evaluate the conservation. Tests were run immediately after the venipuncture (A), after 3-month freezing (− 20 °C), (B) and after 6-month freezing (− 20 °C), (C). In B and C, serum was defrosted at room temperature. Coefficient of Variation (CV) for A vs B, A vs C, and for B vs C was calculated.

### Statistical analysis

All haemato-biochemical parameters, TT4 values and redox status markers were checked for normal distribution, before applying parametric tests. In order to neutralize the possible interference of age, gender, BCS as comparing dROMs, OXY-Adsorbent and Osi values in Group H, I and C, different subgroups were preliminarily identified. The following criteria were adopted: age-subgroups were classified according to AAFP-AAHA guidelines [32], as mature (cats within the age interval of 6–10 years), senior (cats within the age interval of 11–14 years) and geriatric (cats elder then 15 years). Sex-subgroups comprehended male (M), female (F), castrated male (CM) and neutered female (NF) cats. BCS-subgroups were classified according to WSAVA guidelines [28] as under ideal (cats with a BCS of 1–3 out of 9), ideal (cats with a BCS of 5 out of 9) and over ideal (cats with a BCS of 6–9 out of 9). To identify correlations between d-ROMs, OXY-Adsorbent, Osi and covariates considered (age, sex, BCS, selected haemato-biochemical parameters such as RBC, WBC, HCT, Hb, BUN, Crea, Alb, ALT and GLU), a preliminary statistical analysis was performed. A quantile multivariate regression model was applied using the software StataCorp. 2015 (Stata: Release 14. Statistical Software. College Station, TX: StataCorp LP). The statistical significance was set at 5% level (*p* &lt; 0.05). Age, sex and BCS didn’t significantly influenced dROMs, OXY-Adsorbent and Osi values when comparing subgroups of Group H, Group I and Group C (*p* &gt; 0.05; Additional file 1: Table S2). No significant correlation was seen between Albumin, dROMs and OXY, while Albumin and BUN were negatively, but significant correlated with Osi (*p* &lt; 0.05 and *p* &lt; 0.001, respectively; Additional file 1: Table S3). Given the above, a possible interference between d-ROMs, OXY-Adsorbent, Osi and covariates identified was considered irrelevant; then, data were simply compared between hyperthyroid, chronically ill and healthy cats (without subgroups) using ANOVA with Tukey’s multiple comparisons test Statistical analysis was performed using GraphPad Prism 7.04 (GraphPad software, CA, USA). Significance was set at 5% (*p* &lt; 0.05).

## Results

Of the 180 cats assessed during recruitment, 154 were deemed eligible for enrollment in the study, matching all inclusion criteria and having a complete clinical and nutritional history. Furthermore, 30 of the 154 serum samples stored for redox status assessment were discarded before analysis because of flocculation, defrosting or hemolysis, leaving complete data for 124 cats. Signalment of cats included in the three groups and their subgroups are reported in Table 1. Haemato-biochemical parameters, including serum TT4 concentration, are presented in Table 2.

**Table 1:** Signalment and diseases diagnosed in cats included in groups C, I and H

	Group C (39 cats)	Group I (45 cats)	Group H (40 cats)
Age (years)	9.9 ± 2.0 α	10.4 ± 3.3 α	12.9 ± 3.1 β
Sex (%)	5.2 F 46.1 NF 20.5 M 28.2 MC	4.3 F 28.9 NF 6.8 M 60 MC	37.5 NF 62.5 MC
Predominant Breed (%)	89.7 DSH	84.5 DSH	90 DSH
Other breeds (%)	5.1 Sphynx 2.6 Chartreaux 2.6 Devon Rex	8.9 Persian 2.2 British short hair 2.2 Devon rex 2.2 Siamese	5 Persian 2.5 Siamese 2.5 Maine Coon
Body weight (Kg)	4.9 ± 1 α	4.9 ± 1.6 α	4.2 ± 1.2 β
BCS (1–9 scale)	5 ± 1 α	4.8 ± 1.6 α	4 ± 1 β
Diseases diagnosed n and (%)	/	Infectious/Inflammatory 16 (35.6) CKD 14 (31.1) Metabolic 6 (13.3) Neoplastic 8 (17.8) Other 1 (2.2)	Hyperthyroidism

Signalment and diseases diagnosed in cats included in groups C, I and H. Data are expressed as Mean ± Standard deviation or Percentage (%)

*BCS* Body condition score, *DSH* domestic short hair, *M* male, *F* female, *MC* male castrated, *NF* neutered female, *n* = number of cats

Data were compared by ANOVA with Tukey’s multiple comparisons post-test. Different symbols (α, β) indicate differences between groups (*p* &lt; 0.05)

**Table 2:** Haemato-biochemical parameters of groups C, I and H

Selected haemato-biochemical parameters	Group C (39 cats)	Group I (45 cats)	Group H (40 cats)	*Reference range*
TT4 (nmol/L); (μg/dL ± *Sd)*	25.7 nmol/L	28.3 nmol/L	106 nmol/L	*10.3–54*
	2 ± 0.5 μg/dL α	2.2 ± 0.6 μg/dL α	8.3 ± 4.2 μg/dL β	*nmol/L; 8–4.2 μg/dL*
Alb (g/dL)	3.6 ± 0.6 α	3.4 ± 1.1 αβ	3.1 ± 0.6 β	*2.2–4.4*
BUN (mg/dL)	21 ± 4.9 α	51 ± 26 β	33 ± 8.7 α	*10–30*
Crea (mg/dL)	1.2 ± 0.2 α	2.6 ± 2.5 β	1.5 ± 0.8 α	*0.3–1.6*
Glucose (mg/dL)	106 ± 26 α	151 ± 88 β	147 ± 27 β	*70–150*
ALT (IU/L)	75 ± 22 α	118 ± 107 αβ	148 ± 129 γ	*20–100*
RBC × 10^6^ (/uL)	5.9 ± 1.3	6.6 ± 1.4	6.7 ± 1.7	*4.6–10.0*
Hct (%)	36 ± 5	37 ± 6	35 ± 7	*28–49*
WBC × 10^3^ (/uL)	9.9 ± 3.6 α	18.6 ± 9.9 β	10.1 ± 3.3 α	*5.5–19.5*

Selected haemato-biochemical parameters of groups C, I and H. Data are given as Mean ± Standard Deviation

*Alb* albumin, *ALT* alanine-amino transferase, *BUN* blood urea nitrogen, *Crea* creatinine, *Hct* haematocrit, *RBC* red blood cells, *TT4* serum total tetraiodothyroxine, *WBC* white blood cells

Data were compared by ANOVA with Tukey’s multiple comparisons post-test. Different symbols (α, β, γ) indicate differences between groups (*p* &lt; 0.05)

The domestic short-hair breed made up almost 90% of the cats recruited. The predominant gender was the male one in the diseased population (62.5% in group H and 66.8% in group I, respectively), while sex distribution was more homogeneous in the control group. Although mature, senior and geriatric cats were purposefully recruited as controls cats, the hyperthyroid population was significantly older (mean age of 12.9 ± 3.1 years) as compared to healthy and chronically ill cats (9.9 ± 2.0 years and 10.4 ± 3.3 years respectively, *p* &lt; 0.01 and *p* &lt; 0.05; Table 1). Hyperthyroid cats also had significantly lower body weight and body condition score compared to groups C and I (*p* &lt; 0.01 and *p* &lt; 0.05, respectively; Table 1). Diseases diagnosed in cats included in group I are given in Table 1. The predominant illness was a chronic infectious or inflammatory condition (35.6% of all chronically ill cats).

When considering haemato-biochemical parameters, group H had significantly lower serum albumin concentration and higher serum glucose concentration than healthy cats (*p* &lt; 0.05; Table 2) and a greater hepatocellular damage as compared to healthy and diseased cats (*p* &lt; 0.01 and *p* &lt; 0.05, respectively; Table 2). Results of other serum parameters fitted with criteria of inclusion established for the study. For instance, the serum TT4 concentration of group H was significantly higher as compared to groups I and C (*p* &lt; 0.01 and *p* &lt; 0.05, respectively). Chronically ill patients (group I) had significantly higher WBC count as compared to the other two groups (*p* &lt; 0.01), due to the enrollment of patients affected from infectious/inflammatory diseases, and a greater impairment (*p* &lt; 0.05) of renal function due to the inclusion of cats diagnosed with CKD IRIS stage ≥2, (Table 2).

**Fig. 1:**
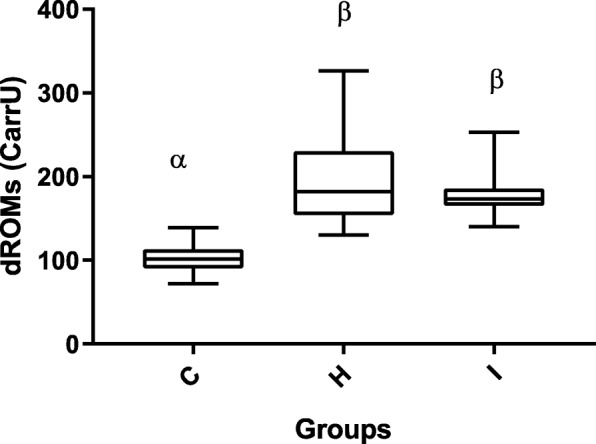
Box-and-whisker plot of dROMs (determinable reactive oxygen metabolites) values in Groups C, H and I. The median is indicated by a horizontal line, the boxes indicate the second and third quartile, and the whiskers include 95% of the data. Different symbols (α, β) indicate differences between groups (*p* &lt; 0.05)

**Fig. 2:**
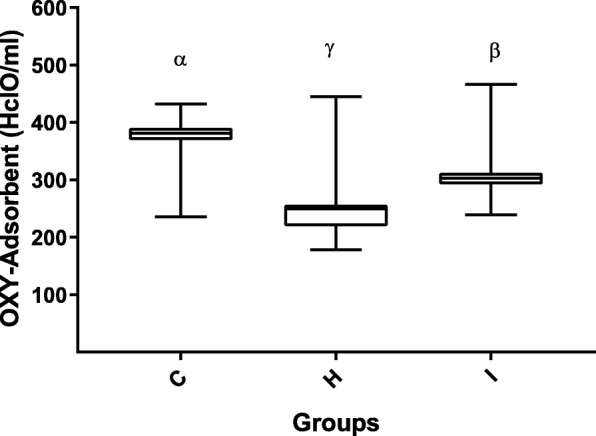
Box-and-whisker plot of OXY-Adsorbent values in Groups C, H and I. The median is indicated by a horizontal line, the boxes indicate the second and third quartile, and the whiskers include 95% of the data. Different symbols (α, β, γ) indicate differences between groups (*p* &lt; 0.05)

**Fig. 3:**
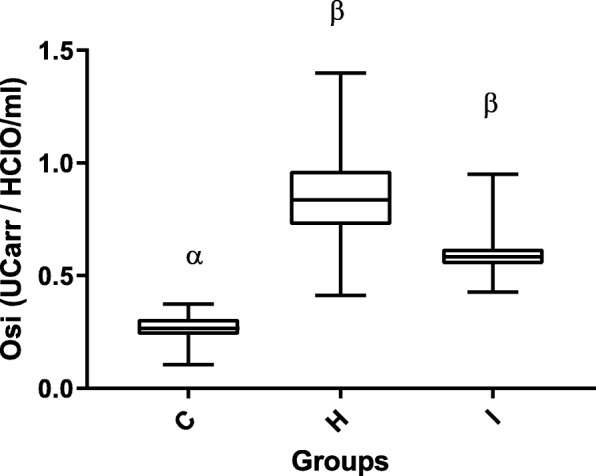
Box-and-whisker plot of OSi (oxidative stress index) values in Groups C, H and I. The median is indicated by a horizontal line, the boxes indicate the second and third quartile, and the whiskers include 95% of the data. Different symbols (α, β) indicate differences between groups (*p* &lt; 0.05)

As for oxidative stress markers, intra-assay and inter-assay coefficients of variation (CV) for dROMs test were 1.91 and 1.72%, respectively, with a coefficient of linear regression (R^2^) of 0.99. Intra-assay and inter-assay CV for OXY-Adsorbent were 1.76 and 1.45 with *R*^2^ = 0.98, (Additional file 1: Table S1). As regard stability after prolonged storage, the CVs for dROMs between group A and B, between group A and C and between group B and C were 3.4, 5.8% and 1.9, respectively. The CVs for OXY-Adsorbent test between group A and B, between group A and C and between group B and C were 3.47, 4.7% and 1.12, respectively. Determinable reactive oxygen metabolites (dROMs) of the hyperthyroid cats (193 ± 47 CarrU) were significantly higher (*p* &lt; 0.001) than those of the healthy cats (103 ± 17 CarrU), and although the dROMs of group I (185 ± 45 CarrU) were lower than in group H, this difference was not significant (Fig. 1). The OXY-Adsorbent test results in the hyperthyroid cats (265 ± 68 μmol HClO/ml) were significantly lower than those in healthy cats (390 ± 83 μmol HClO/ml; *p* &lt; 0.01) and chronically ill cats (306 ± 45 μmol HClO/ml, *p* &lt; 0.05) (Fig. 2). Moreover, the Osi value in group H (0.8 ± 0.2 CarrU/μmol HClO/ml) was significantly higher (*p* &lt; 0.001) than that of the healthy cats (0.3 ± 0.1 CarrU/μmol HClO/ml), and although the Osi value in group H was higher than in group I (0.6 ± 0.1 CarrU/μmol HClO/ml), this difference was not significant (Fig. 3).

## Discussion

Feline hyperthyroidism is recognized as the most common endocrine disease in mature cats. Although its etiopathogenesis remains unclear [2, 3], it resembles human thyrotoxic syndromes [3] in which redox unbalance has been previously described as playing a role in the severity of clinical signs [10, 11] and in idiosyncratic drug reactions [13–15]. Therefore, we assumed that hyperthyroid cats could also develop a redox unbalance, as compared to healthy cats or cats with chronic non-thyroidal illness. The cohort of hyperthyroid cats recruited for the current study was in line with the population of hyperthyroid cats described in the literature. Signalment and haemato-biochemical values of our hyperthyroid population were harmonized with those in recent trials [4, 31], and overlapped with the cohort investigated by Branter et al. [25] who evaluated the antioxidant status of hyperthyroid cats before and after radioiodine treatment. Moreover, the healthy population was quite comparable to the group referred to as A2 (healthy cats with an age ranging from 7 to 12 years) in the study from Castillo et al. [30] and to the control group from Branter et al. [25]. However, age tended to be higher than Castillo et al. [30], with regard to the ill and hyperthyroid group and their results suggest that dROMs values tends to decrease with ageing, which could suggest that oxidative stress is even more pronounced in the hyperthyroid cats then in other categories. The impact of gender (≈50% of F in the control group vs ≈ 40% of F in the ill and hyperthyroid group) could also have skewed the results. However, considering preliminarily results obtained from the regression model as comparing age-subgroups, sex-subgroups and BCS-subgroups, the possible influence of aforementioned covariates seems to be negligible. In the present study, then, redox unbalance was mainly influenced from the status of disease rather from other parameters considered.

Reactive oxygen metabolites (dROMs) and OSi values were higher in hyperthyroid cats than in healthy cats, indicating that thyrotoxicosis may affect the redox balance, as described in humans and laboratory animals [8, 9]. Furthermore, although a significant increase in redox unbalance (increased dROMs and OSi values and decreased OXY-adsorbent test results) was observed also in the cats with other chronic diseases (Group I), the hyperthyroid population (group H) had the greatest impairment of antioxidant defences (i.e., a greater decrease in OXY-Adsorbent values). This result seems to be in contrast with the study from Branter et al. [25] where urinary 8-isoprostanes (markers of oxidative stress) were significantly increased in hyperthyroid cats, even though circulating blood antioxidants were not depleted [25]. Thirty of the 44 hyperthyroid cats enrolled in the aforementioned trial, however, had been treated previously with methimazole, and had discontinued the drug a median of 12.5 days before evaluation. Moreover, only a small number of cats had documented prior idiosyncratic adverse reactions. Given the above, it is possible that the short discontinuation interval together with the low-rate occurrence of methimazole-related side effects had interfered with their antioxidant status.

Based on our findings, hyperthyroid cats may be specifically identified as possible candidates for nutritional supplementation with antioxidants during medical treatment for hyperthyroidism, with the dual goal of reducing the gap between overproduction of free radicals (due to the hypermetabolic state) and the decreased serum antioxidant status. The decrease in serum antioxidant levels in our cohort could be related to either progressive exhaustion of serum antioxidant capacity due to chronic challenge or reduced absorption/increased loss of antioxidant compounds through the small bowel and kidneys. It would be useful to evaluate the activity of endogenous antioxidant enzymes (e.g. SOD, CAT and GPx) in such a group in a future trial. Our present results are not able to elucidate the mechanism underlying the redox unbalance in hyperthyroid cats.

The general increase in oxidative stress markers during chronic illness detected in the current study is shared by previous studies [18–24], but a reliable comparison with the diseased population from literature could not be performed due to heterogeneity in signalment and type of illnesses considered.

Moreover, we used different assays to quantify free radical production. This constitutes a crucial aspect of redox balance assessment in veterinary medicine: there is no unique, standardized method that describes oxidative stress and antioxidant status in feline disease [27], which hampers an accurate comparison across trials and the identification of species-specific reference ranges [30, 31, 33, 34]. To date, only one short communication has been published in which dROMs and OXY-Adsorbent tests to assess oxidative stress and antioxidant status in healthy cats were described [30], while studies generally report findings in other species like dog and humans [33, 34]. Our data, however, seem to support the use of dROMs, OXY-Adsorbent and OSi as possible markers of redox status in the feline species, also after prolonged storage of serum samples at − 20 °C. However, our data support the use of dROMs, OXY-Adsorbent and OSi as possible markers of redox status in the feline species, even after storage of serum samples at − 20 °C for 3 months.

Pasquini et al. [31] found that dROMs values ranged from 56 to 91 CarrU in Labrador dogs, while a mean dROMs value of 396 CarrU and a mean OXY-Adsorbent value of 416 μmol HClO/ml were reported in healthy humans [34]. This means that physiological levels of free radicals in feline plasma (a mean dROMs of 103 CarrU in our healthy population and a mean of 126 CarrU in the population [cats &gt; 7 years] studied by Castillo et al.) [30], fall somewhere in between the normal levels in dogs and humans. Species-specific differences in the mechanisms counteracting oxidative stress damage are likely to exist and need to be considered when developing translational models of diseases.

The limitations of the present study are the non-standardized diet and the non-standardized definition of disease type in group I that could have interfered with redox status. Despite preliminarily data obtained from the multivariate analysis, the lack of age matching, sex-matching and BCS-matching groups could also represent a possible confounding factor, still. Further studies in a more controlled setting are warranted to confirm our findings and to address these possible biases. Moreover, the study of redox unbalance before and after hyperthyroidism treatment would also have strengthened our findings. Given the above, a double-blind, placebo controlled, randomized clinical trial evaluating the effect of methimazole alone or in combination with antioxidant supplementation is ongoing at the time of manuscript’s drafting.

## Conclusion

The present study showed for the first time that, as previously described in hyperthyroid humans, redox unbalance can be determined in hyperthyroid cats and that cats may provide a spontaneous animal model for oxidative stress-related studies linked to thyrotoxicosis. Furthermore, our findings could also have a potential clinical application, where early diagnosis and treatment of the disease, as well as reducing drug-related side effects, are the key-factors in successful treatment outcome. Given the role of redox unbalance in the course of FH, clinical trials investigating the potential positive effects of antioxidant supplementation in hyperthyroid cats are desirable.

## Additional file

Additional file 1: **Table S1.** Validation data for dROMs and OXY-Adsorbent assays on feline serum collected by authors and their comparison with DIACRON® and Pasquini et al., 2008. **Table S2.** Preliminary statistical analysis with the quantile multivariate regression model. Correlations between d-ROMs, OXY-Adsorbent, Osi and age, gender and BCS. **Table S3.** Preliminary statistical analysis with the quantile multivariate regression model. Correlations between d-ROMs, OXY-Adsorbent, Osi and selected haemato-biochemical parameters (BCS, RBC, WBC, Hct, Hb, BUN, Crea, Alb, ALT and GLU). (DOCX 23 kb)

## Abbreviations

AAFP-AAHA: American Association of Feline Practitioners (AAFP) and American Animal Hospital Association (AAHA)
ALB: Albumin
ALT: Alanine-amino transferase
BCS: Body condition score
BUN: Blood urea nitrogen
CarrU: Carratelli Units
CKD: Chronic kidney disease
CREA: Creatinine
CV: Coefficient of variation
dROMs: Determinable reactive oxygen metabolites
DSH: Domestic shorthair
F/NF: Female/neutered female
FH: Feline hyperthyroidism
GLU: Glucose
Hb: Hemoglobin
Hct: Hematocrit
ISFM: International Society of Feline Medicine
M/MC: Male/castrated male
OS: Oxidative stress
OSi: Oxidative stress index
RBC: Red blood cells
T4: Serum total tetraiodothyroxine
WBC: White blood cells
