# Supplementary material for: Redox unbalance in the hyperthyroid cat: a comparison with healthy and non-thyroidal diseased cats
Source: BMC Vet Res. 2019 May 8;15:136. doi: 10.1186/s12917-019-1896-7 (PMC6505105; doi:10.1186/s12917-019-1896-7)
Supplement: Supplementary file 1 — Table S1. Validation data for dROMs and OXY-Adsorbent assays on feline serum collected by authors and their comparison with DIACRON® and Pasquini et al., 2008. Table S2. Preliminary statistical analysis with the quantile multivariate regression model. Correlations between d-ROMs, OXY-Adsorbent, Osi and age, gender and BCS. Table S3. Preliminary statistical analysis with the quantile multivariate regression model. Correlations between d-ROMs, OXY-Adsorbent, Osi and selected haemato-biochemical parameters (BCS, RBC, WBC, Hct, Hb, BUN, Crea, Alb, ALT and GLU). (DOCX 23 kb) [file 12917_2019_1896_MOESM1_ESM.docx]

**Additional files**

**Table S1. Validation data for dROMs and OXY-Adsorbent assays on feline serum collected by authors and their comparison with DIACRON® and Pasquini et al., 2008**

|  | **dROMs assay on feline serum (author’s data)** | **dROMs assay on human serum (DIACRON® data)** | **dROMs assay on dog serum (Pasquini et al., 2008 [31])** |
| --- | --- | --- | --- |
| *within-run precision (intra-assay CV)* | *N=12 CV%=1.91%* | *N=20 CV%=1.90* | *N=4 CV%=3.7* |
| *day- to day precision (inter-assay CV)* | *N=12 CV%=1.72%* | *N=20 CV%=2.05* | *N=80 CV%=2.05* |
| *linearity* | *R2=0.99* | *R2=0.99* | *R2=0.95* |
|  | **OXY-Adsorbent assay on feline serum (author’s data)** | **OXY-Adsorbent assay on human serum (DIACRON data)** | **OXY-Adsorbent assay on dog serum (Pasquini et al., 2008)** |
| *within-run precision (intra-assay CV)* | *N=12 CV%=1.91%* | *N=20 CV%=1.90* | *na (BAP was tested instead of OXY- Adsorbent assay)* |
| *day- to day precision (inter-assay CV)* | *N=12 CV%=1.72%* | *N=20 CV%=2.05* | *na* |
| *linearity* | *R2=0.99* | *R2=0.99* | *na* |

*N=number of samples; CV=Coefficient of Variation; R^2^= Coefficient of Regression; na= not assessed.*

*Pasquini et al., 2008= see reference 31 in the Manuscript*

**Table S2. Preliminary statistical analysis with the quantile multivariate regression model. Correlations between d-ROMs, OXY-Adsorbent, Osi and age, gender and BCS.**

|  | **Correlation** | **P>t** |
| --- | --- | --- |
| **dROMs, OXY-Adsorbent and Osi** | **Age-subgroups of Group C vs Age subgroups of Group I** | No correlation (P>t 0.05) |
|  | **Age-subgroups of Group C vs Age subgroups of Group H** | No correlation (P>t 0.05) |
|  | **Age-subgroups of Group I vs Age subgroups of Group H** | No correlation (P>t 0.05) |
| **dROMs, OXY-Adsorbent and Osi** | **Sex-subgroups of Group C vs Sex-subgroups of Group I** | No correlation (P>t 0.05) |
|  | **Sex-subgroups of Group C vs Sex-subgroups of Group H** | No correlation (P>t 0.05) |
|  | **Sex-subgroups of Group I vs Sex-subgroups of Group H** | No correlation (P>t 0.05) |
| **dROMs, OXY-Adsorbent and Osi** | **BCS-subgroups of Group C vs BCS subgroups of Group I** | No correlation (P>t 0.05) |
|  | **BCS-subgroups of Group C vs BCS subgroups of Group H** | No correlation (P>t 0.05) |
|  | **BCS-subgroups of Group I vs BCS subgroups of Group H** | No correlation (P>t 0.05) |

*Age-subgroups were classified according to AAFP-AAHA guidelines [32], as mature (cats within the age interval of 6-10 years), senior (cats within the age interval of 11-14 years) and geriatric (cats elder then 15 years). Sex-subgroups comprehended male (M), female (F), castrated male (CM) and neutered female (NF) cats. BCS-subgroups were classified according to WSAVA guidelines [28] as under ideal (cats with a BCS of 1-3 out of 9), ideal (cats with a BCS of 5 out of 9) and over ideal (cats with a BCS of 6-9 out of 9).* *Statistical analysis was performed using the software StataCorp. 2015 (Stata: Release 14. Statistical Software. College Station, TX: StataCorp LP. Statistical significance set at 5% level (p < 0.05)*

**Table S3. Preliminary statistical analysis with the quantile multivariate regression model. Correlations between d-ROMs, OXY-Adsorbent, Osi and selected haemato-biochemical parameters (BCS**, **RBC, WBC, Hct, Hb, BUN, Crea, Alb, ALT and GLU).**

|  | **Correlation** | **Coefficient** | **Std.E** | **t** | **P>t** | **95% CI** | **Group** |
| --- | --- | --- | --- | --- | --- | --- | --- |
| **dROMs** | Crea | 11.5 | 5.6 | 2.04 | 0.044 | 0.3 | 22.6 |
| **OXY-Adsorbent** | No correlation (P>t 0.05) | | | | | | |
| **Osi** | Alb | -0.05 | 0.02 | -2.41 | 0.019 | -0.09 | -0.01 |
|  | BUN | -0.01 | 0.00 | -4.31 | 0.000 | -0.01 | 0.00 |
|  | Crea | 0.08 | 0.02 | 3.63 | 0.001 | 0.04 | 0.13 |

*Alb: albumin, ALT: alanine-amino transferase, BUN: blood urea nitrogen, Crea: creatinine, GLU: glucose; Hct: haematocrit, RBC: red blood cells, WBC: white blood cells.* *Statistical analysis was performed using the software StataCorp. 2015 (Stata: Release 14. Statistical Software. College Station, TX: StataCorp LP). Statistical significance set at 5% level (p < 0.05)*
